# Characterization of finger millet extracts and evaluation of their nematicidal efficacy and plant growth promotion potential

**DOI:** 10.1002/pei3.70006

**Published:** 2024-08-20

**Authors:** Khaoya Martine Chrisantus, Chepkwony Sarah, Lusweti Dorcas, Rose Chepchirchir Ramkat, Chrispus O. A. Oduori, Njira Njira Pili

**Affiliations:** ^1^ Department of Biological Sciences Moi University Eldoret Kenya; ^2^ Department of Chemistry and Biochemistry Moi University Eldoret Kenya; ^3^ Kenya Agricultural and Livestock Research Organization (KALRO) Kisumu Kenya

**Keywords:** beneficial microbes, biological control, biopriming, botanicals, Kenya, Okhale‐1, root lesion nematodes, *wimbii*

## Abstract

Plant‐parasitic nematodes pose a significant threat to finger millet crops, potentially causing yield reduction of up to 70%. Extracts derived from finger millet varieties contain potent bioactive compounds that can mitigate nematode damage and promote plant growth. This study aimed at isolating and characterizing bioactive compounds from the finger millet varieties Ikhulule, Okhale‐1, and U‐15; evaluating the impact of Ikhulule and U‐15 extracts on the mortality of the root lesion nematode *Pratylenchus vandenbergae*; assessing the growth promotion effects of Ikhulule and U‐15 extracts on the finger millet variety Okhale‐1; and determining the efficacy of these extracts in managing plant‐parasitic nematodes under greenhouse conditions. Extracts were obtained from both leaves and roots and tested in vitro for nematode mortality and in vivo for growth promotion and nematode control. The results showed that finger millet extracts exhibited strong nematicidal properties in vitro, achieving a mortality rate of up to 98% against *P. vandenbergae* nematodes. Applying these extracts to finger millet shoots significantly reduced nematode populations in both soil and roots and decreased the reproductive factor to below one (1), indicating an effective nematode control. The study attributes the enhanced nematicidal effects of finger millet extracts to their bioactive compounds, particularly dodecanoic acid, phytol, 1,1,4a‐trimethyl‐6‐decahydro naphthalene, 2,3‐dihydro‐benzofuran, 2‐methoxy‐4‐vinylphenol and ethyl ester, and hexadecanoic acid. These findings suggest that finger millet‐derived extracts offer a natural solution for nematode management and broader agronomic benefits, ultimately contributing to overall plant health and productivity.

## Graphical abstract

Finger millet has a diverse set of phytochemicals. These chemicals can kill nematodes directly after 72 hours of soaking. Indirectly, phytochemicals can enhance the weight of seeds, indicating that extract‐treated plants have larger seeds than the control plants.

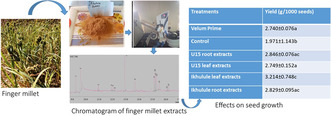

## 1 INTRODUCTION

Finger millet (*Eleusine coracana* L.) is an important staple food crop in Kenya, particularly in semiarid regions. Finger millet is primarily used to produce flour for traditional dishes like ugali and beverages such as uji and busaa (local alcoholic drink). Its high levels of calcium, iron, and dietary fiber make it nutritionally valuable, especially for children, pregnant women, and those with specific dietary needs. Additionally, finger millet straws serve as important animal fodder, providing nutrition for livestock and income for smallholder farmers. As a drought‐tolerant and versatile crop, finger millet significantly enhances food and nutritional security in Kenya (Onyango, 2016; Waweru et al., 2023).

Finger millet production has been declining over the years (Onyango, 2016) due to various factors, including diseases caused by plant‐parasitic nematodes (Chung et al., 2020; Mbinda et al., 2021; Singh et al., 2021). Several nematodes, including the newly identified species *Rotylenchus wimbii* and *Pratylenchus vandenbergae*, have been isolated from finger millet (Singh et al., 2021; Wanjau et al., 2023). Research indicates that *P. vandenbergae* is highly pathogenic to finger millet, causing yield losses of up to 70% (Waweru et al., 2023). This nematode has a high multiplication rate (a) of 32.39 and a maximum population density (M) of 18.83 per season. The tolerance limit of finger millet to *P. vandenbergae* is 0.59 nematodes per gram of soil, above which, significant damage occurs, requiring effective management strategies.

Research into the management of plant‐parasitic nematodes in finger millet has been ongoing, focusing on the potential of biopriming using beneficial microbes and botanicals. Preliminary findings indicate that finger millet root exudates may inhibit the hatching of juveniles of the root‐knot nematode (RKN) *Meloidogyne javanica* from eggs, suggesting the presence of secondary metabolites (C. Wanjau, unpublished data).

Plants accumulate natural metabolites that serve various functions, acting as repellents, attractants, hatch stimulants, or nematotoxicants, thereby providing the plant with a robust defense strategy (Waweru et al., 2022). The use of plant compounds like flavonoids, alkaloids, and saponins as defense mechanisms in plants is well documented (Abdullah et al., 2023). Compounds from plants, such as *Tagetes* spp., mustard, canola, and cassava, are nematicidal against nematodes. Finger millets contain comparable phytochemicals with known antimicrobial effects.

The objectives of this study were to isolate and characterize bioactive compounds from the finger millet varieties Ikhulule, Okhale‐1, and U‐15; evaluate the impact of Ikhulule and U‐15 extracts on the mortality of nematodes; assess the growth promotion effects of Ikhulule and U‐15 extracts on the finger millet variety Okhale‐1; and determine their efficacy in managing plant‐parasitic nematodes under greenhouse conditions. The use of botanicals in biopriming supports sustainable agriculture, by proving environmentally friendly and long‐term solutions for nematode management. Bioassays using phytochemicals from finger millet contribute to the sustainable nematode management by reducing over‐dependency of chemical pesticides.

## 2 MATERIALS AND METHODS

### 2.1 Finger millet varieties

Three finger millet varieties, Ikhulule, Okhale‐1, and U‐15, were cultivated on a research farm at Moi University, located at longitudes 35°17′78E, latitudes 00°17′14N, and an elevation of 2224 m above sea level, following farmer practices. Three months post‐germination, the leaves and roots of the plants were harvested, cut into small pieces, and air‐dried under the shade for 3 weeks. The dried plant materials were then ground into a fine powder (1 mm) using a NutriBullet® 600 Series electric grinder (Capbran Holdings, LLC Los Angeles, CA, USA) and stored in brown khaki paper bags at room temperature until further use (Mugomeri et al., 2014).

### 2.2 Extraction of phytochemicals by maceration method

The maceration method was used to extract phytochemicals from the finger millet plants. Each sample, consisting of 50 g of leaf or root powder, was soaked separately in 400 mL of ethanol and kept in darkness for 72 h with occasional shaking. Subsequently, filtration was carried out using Whatman No. 1 filter paper. The resulting filtrates were then concentrated to dryness using a Hahnvapour HS‐2005S vacuum rotary evaporator (Hahnshin S & T Limited, Korea) at 40°C under reduced pressure. The dried extracts were stored in sealed vials at 4°C pending further analyses. The percentage yield of the extracts was calculated using the following formula:

(1) $$\text{Extracted yield}\%=\frac{\text{Weight of the}\;\mathrm{dry}\;\text{extract}\;\left(g\right)}{\text{Weight of the sample used for extraction}\;\left(g\right)}\times 100$$

### 2.3 Phytochemical screening of the crude extracts

The crude extracts were screened for phytochemicals to identify the presence of secondary metabolites, as detailed in Table 1.

**TABLE 1:** Procedure of determining secondary metabolites from finger millet extracts.

Secondary metabolite	Procedure	Confirmatory observations	Reference
Phenols	A few drops of 5% ferric chloride solution +2 mL of extracts followed by gentle shaking	Formation of red/dark green to bluish black color	Mansoori et al. (2020), Bhati et al. (2021)
Glycosides	1.5 mL of glacial acetic acid +1 mL of extract+1 drop of 5% ferric chloride +2 drops of concentrated sulfuric acid	Formation of reddish‐brown color	Prasanth et al. (2016)
Tannins	1 mL of the extract +3 mL of distilled water +3 drops of 10% ferric chloride solution, followed by gentle shaking	Color change from green to brown	Mansoori et al. (2020), Bhati et al. (2021)
Saponins	5 mL of extract +5 mL of distilled water, followed by vigorous shaking for 15 min, allowing to stand for 10 min	Formation of a foam	Mansoori et al. (2020), Bhati et al. (2021)
Alkaloids	A few drops of Wagner's reagent solution +3 mL of extract, followed by gentle shaking	Formation of a brown‐reddish precipitate	Bhati et al. (2021)
Terpenoids	3 mL of concentrated sulfuric acid (H_2_SO_4_) + 0.2 g of extract in 2 mL of chloroform	Formation of a gray‐colored solution	Mansoori et al. (2020), Bhati et al. (2021)
Flavonoids	2 mL of the extracts +2 mL of 2% sodium hydroxide solution + a few drops of dilute hydrochloride acid	Formation of a bright yellow color	Bhati et al. (2021)

### 2.4 Characterization of the extracts by gas chromatography–mass spectrometry

The extracts from the Ikhulule, Okhale‐1, and U‐15 varieties were concentrated using solid‐phase extraction (SPE). An Agilent Bond Elute C18, 3 cc, was mounted on an Agilent SPE manifold and conditioned with 3 mL of methanol. This was equilibrated with 3 cc of HPLC‐grade water and then 100 mL of sample was added. The cartilage was washed with a 5:95 methanol:water solution and dried in a stream of air for 10 min. The analytes were then eluted with 3 mL of methanol and concentrated to near dryness using a MiVac DNA concentrator. Later, the analytes were reconstituted with 1 mL of methanol, filtered through 2 mL of 0.45 μM syringe filter into Agilent Vials, and analyzed using a Shimadzu GC‐MS QP201SE fitted with a BPX5 column (length, 30 m; thickness, 0.25 μm; internal diameter, 0.25 mm).

Extracts were injected in split mode (1:10) at a temperature of 20°C. The column oven temperature was adjusted to 60 DC, and the temperature program used to separate compounds starting from 60 DC, was ramped at 15 DC/min to 215 DC, held for 1 min, increased to 280 DC at a rate of 3 DC/min, and held for 3 min. The interface temperature was set to 250 DC, the ion source was set to 200 DC, and the solvent cutoff duration was 4.5 min. The mass spectrometer was operated in scan mode for masses ranging from 35 to 550 Hz. The compounds were tentatively identified using the NIST library 14 based on their mass‐to‐charge ratios (m/z), fragmentation patterns, and comparison with published spectroscopic data.

### 2.5 Culturing of the root lesion nematode *Pratylenchus vandenbergae*

Isolates of *P. vandenbergae*, originally isolated from finger millet plants, were cultured on carrot discs following the procedures described by Coyne et al. (2014). A mixture of life stages was extracted from the carrot discs after 4 months of cultivation and stored in falcon tubes awaiting the next step. Afterward, the inoculum was concentrated, and the density was estimated at 1500 nematodes per 3‐L pot.

### 2.6 In vitro nematicidal activity of extracts against *P. vandenbergae*

The nematicidal activity of the finger millet extracts and the commercial nematicide velum prime® was performed through direct contact bioassays at room temperature in a laboratory. The leaf and root extracts of Ikhulule and U‐15 were dissolved in distilled water to form a 20‐mg/mL stock solution, which was subsequently diluted to a concentration of 1 mg/mL. The in vitro bioassay activity of the two varieties was evaluated against mixed stages of *P. vandenbergae* (Ghareeb et al., 2019). The mortality rate of 50 nematodes was determined by adding 0.5 of 1 mg/mL extract in a 2‐mL Eppendorf micropipette (Eppendorf®, Hamburg, Germany). The nematodes were soaked in 0.5 mL of distilled water (negative control) or velum prime® (positive control) for the control experiments. Thereafter, all the treatments were incubated at 25 ± 2°C for 24, 48, and 72 h. To assess whether the nematodes were dead or alive, 1 N sodium hydroxide solution was added to the solution followed by a 24‐h soaking in distilled water (Khan et al., 2016). Nematodes were considered dead if they remained straight or slightly curved after being probed with a fine needle under a light microscope (Xiang & Lawrence, 2015). The percentages of dead and live nematodes were recorded, and the percentage mortality was calculated following the protocol of Khan et al. (2016). This experiment was performed twice with three replicates for each treatment. To estimate the time required for nematicidal activity by the extracts, root lesion nematode (RLN) mortality was performed at different time intervals of 24, 48, and 72 h.

### 2.7 Pot bioassays in a greenhouse

The experiment was conducted on the Okhale‐1 finger millet variety known for its high yield and tolerance to drought, striga weed, and blast disease. The seeds were surface sterilized with 70% ethanol for 1 min and 1% sodium hypochlorite solution for 10 min. They were then pre‐germinated on moistened filter papers for 5 days before being transplanted into 4 cm diameter plastic pots (one seedling per pot) containing 3 kg of autoclaved sterilized sand and agricultural soil in a ratio of 1:2. The greenhouse conditions were maintained at temperatures between 27 and 32°C, with a 12‐h light regime. The plants were watered every 2 days with 200 mL of water and fertilized weekly with Rosasol (N: P: K, 30:10:10; Twiga Chemical Industries, Ltd.) commercial fertilizer (Waweru et al., 2023). This experiment had six treatments, each replicated eight times as described in Table 2.

**TABLE 2:** Summary of treatments and the targeted observations.

S/No	Treatment (T)	Scale
1	T1: plants + *Pratylenchus vandenbergae* + U‐15 leaf extracts	Present (+), moderately present (++), and highly present (+++)
2	T2: plants + *P. vandenbergae* + Ikhulule root extracts	
3	T3: plants + *P. vandenbergae* + U‐15 root extracts	
4	T4: plants + *P. vandenbergae* + Ikhulule leaf extracts	
5	T5: plants + *P. vandenbergae* + distilled water (negative control)	
6	T6: plants + *P. vandenbergae* + velum prime® (positive control).	

Twelve days after transplanting, the shoots of the finger millet plants were sprayed with the extracts (1 mg/mL), velum prime® (0.5 mg/mL) (positive control), or distilled water (negative control). One week later, the plants were inoculated with 1500 mixed stages of *P. vandenbergae* and sprayed again with the extracts, velum prime®, or distilled water at the same concentrations as described above. The experiment was arranged in a randomized complete design (CRD) with eight replicates per treatment. Sixteen weeks after nematode inoculation, plant height, number of tillers, and the fresh weights of roots, shoots, panicles, and dry grain (total and 1000‐seed weight) were measured per pot.

The final population density (*Pf*) of the nematodes was estimated in soil and roots at harvest. The soil was thoroughly mixed, and 300 cm^3^ per pot was used to extract nematodes using Baermann's method (Hooper et al., 2005). Roots from each plant were washed to remove soil debris and cut into small pieces (approximately 1 cm), and a 5 g subsample was taken for nematode extraction using the same method. The total number of nematodes was established from roots and soil after 48 h following the protocol of Wanjau et al. (2023). The reproduction factor of *P. vandenbergae* was calculated using the following formula:

(2) RF=Pf/Pi

where *Pf* = final nematode population and *Pi* = initial nematode population.

### 2.8 Statistical analysis

The in vitro data on the number of dead and live nematodes, along with the final nematode population in a pot experiment, plant height, number of tillers, fresh weight (g) of shoots roots and panicle, and dry grain weight (total and weight of 1000 seeds), were analyzed using SPSS statistical software version 25.0.0. Subsequently, the Tukey post hoc test was applied to identify any significant differences among treatments at a probability level of *p* ≤ .05.

## 3 RESULTS

### 3.1 Percentage yields of finger millet extracts

The yields of the finger millet extracts varied among the Ikhulule, Okhale‐1, and U‐15 varieties, with yield ranges of 1.56%–2.68%, 0.56%–3.12%, and 0.58%–2.10%, respectively. Notably, the yields from the roots were consistently lower than those from the leaves, a trend observed across all millet varieties in the study as detailed in Table 3.

**TABLE 3:** Yields of extracts from the finger millet varieties Ikhulule, Okhale‐1, and U‐15.

Varieties	Ikhulule		Okhale		U‐15	
	LE	RE	LE	RE	LE	RE
Yield % (w/w)	2.68	1.56	3.12	0.56	2.10	0.58

Abbreviations: LE, leaf extracts; RE, root extracts.

### 3.2 Phytochemical composition of finger millet extracts

Seven secondary metabolites were detected using a scale of present (+), moderately present (++), and highly present (+++). Phenols, glycosides, and tannins were moderately present in both leaf and root extracts. Flavonoids were highly present in the leaf extracts of Ikhulule, moderately present in the leaf extracts of Okhale‐1, and U‐15, and present in the root extracts of Ikhulule, Okhale‐1, and U‐15. Alkaloids were highly present in the leaf extracts of all finger millet varieties and moderately present in the root extracts. Terpenoids were moderately present in the leaf extracts of all varieties and present in the root extracts. Saponins were highly present in the root extracts of Ikhulule, moderately present in the root extracts of Okhale‐1 and U‐15, and present in the leaf extracts of all varieties. Collectively, leaf extracts contained moderate to high levels of phenolic compounds, glycosides, tannins, flavonoids, alkaloids, and terpenoids, while root extracts contained present to moderate levels of these compounds (Table 4).

**TABLE 4:** Secondary metabolites from the finger millet varieties Ikhulule, Okhale‐1, and U‐15.

Phytochemicals	Ikhulule		Okhale‐1		U‐15	
	Leaf extract	Root extract	Leaf extract	Root extract	Leaf extract	Root extract
Phenolics	++	++	++	++	++	++
Glycosides	++	++	++	++	++	++
Tannins	++	++	++	++	++	++
Flavonoids	+++	+	++	+	++	+
Alkaloids	+++	++	+++	++	+++	++
Terpenoids	++	+	++	+	++	+
Saponins	+	+++	+	++	+	++

Abbreviations: +, present; ++, moderate; +++, highly present; —, absence.

### 3.3 Gas chromatography‐mass spectrometry analysis of finger millet extracts

Gas chromatography–mass spectrometry (GC‐MS) analysis of the finger millet extracts revealed a rich and complex spectrum of secondary metabolites. The analysis identified over 184 distinct chromatographic peaks. Among these peaks, 49 compounds were consistently detected across multiple plant extracts. Using advanced library matching techniques, these 49 peaks were accurately matched to 41 unique compounds from various chemical classes. Among the identified compounds, 6 were important due to their known biological activities. These compounds included dodecanoic acid, phytol, 1,1,4a‐trimethyl‐6‐decahydro‐naphthalene, 2,3‐dihydro‐benzofuran, 2‐methoxy‐4‐vinylphenol and ethyl ester, and hexadecanoic acid (Table 5).

**TABLE 5:** Major compounds identified from the finger millet extracts through GC‐MS.

S/No	Tentative compound	Retention (min)	Molecular weight	Percent	Genotypes where the extract was present
1	Dodecanoic acid	9.632	235	4.71	Ikhulule, U‐15, and Okhale‐1
2	Phytol	15.320	296	0.41	Ikhulule, U‐15, and Okhale‐1
3	1,1,4a‐trimethyl‐6‐decahydro naphthalene	18.698	272	1.19	Ikhulule, U‐15, and Okhale‐1
4	2,3‐dihydro‐benzofuran	6.575	121	0.51	Ikhulule and U‐15
5	2‐Methoxy‐4‐vinylphenol	7.519	150	0.26	Ikhulule and U‐15
6	Ethyl ester, hexadecanoic acid	13.695	285	15.61	Ikhulule, U‐15 and Okhale‐1

### 3.4 Effects of finger millet extracts on nematode mortality

To determine the toxicity of finger millet extracts on nematodes, in vitro bioassays were performed at different time points (24, 48, and 72 h) and percentage mortality was recorded. Within 24 h, Ikhulule leaf extracts killed 15%–45% of the nematodes, and after 72 h, the mortality rate increased up to 68%. During the same period, the commercial nematicide velum prime® killed between 54% and 83% of the nematodes after 24 h and 89%–97% after 72 h. The Ikhulule root extracts killed 57%–84% of the nematodes after 24 h and 89%–98% after 72 h. Similarly, U‐15 leaf extracts killed 24%–51% of the nematodes after 24 h of soaking and 77%–91% after 72 h. The root extract killed between 61% and 71% of the nematodes after 24 h, reaching a high percentage of 87%–89% after 72 h. The commercial nematicide velum prime® killed more than 98% of the nematodes after 72 h (Table 6).

**TABLE 6:** Effects of finger millet extracts on nematode mortality.

Experiment 1 (% mortality)				Experiment 2 (% mortality)		
Ikhulule leaf extract						
Treatment	24 h	48 h	72 h	24 h	48 h	72 h
Control	2.00 ± 1.15a	4.00 ± 1.15d	9 ± 1.76 g	2.00 ± 1.15a	4.00 ± 1.15d	7 ± 1.76 g
Velum prime®	82.00 ± 3.46c	91 ± 1.76f	97 ± 1.76i	54.00 ± 4.16c	63 ± 2.90f	89 ± 6.35i
Extract	45 ± 5.81b	57 ± 1.76e	69 ± 1.76 h	15.33 ± 1.76b	23.00 ± 3.60e	34.00 ± 1.15 h
Ikhulule root extract						
Control	2.00 ± 1.15a	4.00 ± 1.15d	6.00 ± 1.15 g	4.00 ± 1.15a	6.00 ± 1.15a	8.00 ± 1.15 g
Velum prime®	83 ± 2.90c	88.00 ± 1.15f	97 ± 1.76 h	55 ± 5.81b	73 ± 4.05b	94.00 ± 3.05 h
Extract	57 ± 8.11b	75 ± 2.90e	89 ± 1.76 h	84.00 ± 3.05c	89 ± 1.76c	98.00 ± 1.15 h
U‐15 leaf extract						
Control	2.00 ± 1.15a	6.00 ± 1.15d	6.00 ± 3.05 g	3 ± 1.76a	7 ± 3.52d	12.00 ± 1.15 g
Velum prime®	73 ± 1.76c	84.00 ± 3.46f	98.00 ± 1.15i	79 ± 1.76c	89 ± 2.40f	97 ± 1.67i
Extract	24.00 ± 2.30b	47 ± 4.80e	77 ± 1.76 h	29 ± 2.90b	51 ± 4.05e	91 ± 2.40 h
U‐15 root extract						
Control	4.00 ± 1.15a	7.33 ± 1.76d	8.67 ± 1.76 g	5.00 ± 1.52a	7.00 ± 1.73d	14.67 ± 1.45 g
Velum prime®	77 ± 1.76c	85 ± 1.76f	98.00 ± 1.15i	78.00 ± 2.30c	84 ± 2.02f	97 ± 2.40i
Extract	61 ± 4.05b	73 ± 4.67e	87 ± 2.90 h	63 ± 4.37b	71 ± 2.40e	84 ± 2.33 h

*Note*: The values are the means of eight replicates ± SEs. Means followed by the same letter(s) are not significantly different according to the Tukey HSD test (*p* ≤ .05).

### 3.5 Effects of finger millet extracts on plant growth promotion and nematode control

To assess the ability of the extracts to enhance plant growth and indirectly reduce plant‐parasitic nematodes in the finger millet variety Okhale‐1, the shoots of young plants were sprayed with the extracts and inoculated with the nematodes. Most of the plant growth characteristics such as the number of tillers, fresh roots, shoots, panicles, and total dry grains weight were not significantly different from both the negative and positive controls. However, the weight of 1000 seeds of extract‐treated plants was significantly higher than those of control plants (Table 7).

**TABLE 7:** Effect of finger millet extracts on plant growth promotion.

Treatment	Tiller	Fresh shoot weight	Fresh root weight	Fresh panicle weight	Dry seeds weight	Dry weight of 1000 seeds
Control	7.10 ± 1.15a	118.23 ± 15.83a	83.68 ± 11.21a	55.45 ± 2.43a	21.02 ± 0.56a	1.971 ± 1.143a
Velum prime	9.37 ± 0.94a	151.07 ± 8.21a	110.09 ± 7.84a	50.67 ± 4.17a	21.15 ± 1.77a	2.740 ± 0.076b
Ikhulule leaf extract	8.00 ± 0.75a	117.48 ± 8.46a	99.41 ± 11.20a	44.38 ± 3.69a	20.27 ± 1.56a	3.214 ± 0.748c
Ikhulule root extract	9.12 ± 0.54a	140.98 ± 13.65a	97.48 ± 13.24a	52.05 ± 3.41a	22.73 ± 1.48a	2.829 ± 0.095bc
U‐15 leaf extract	7.00 ± 1.15a	108.37 ± 15.02a	97.16 ± 13.71a	50.21 ± 4.95a	19.22 ± 3.42a	2.749 ± 0.152b
U‐15 root extract	8.62 ± 0.70a	123.10 ± 15.32a	89.18 ± 6.74a	49.17 ± 4.02a	21.39 ± 1.95a	2.846 ± 0.076bc

*Note*: The values are the means of eight replicates ± SEs. Means followed by the same letter(s) are not significantly different according to the Tukey HSD test (*p* ≤ .05).

**FIGURE 1:**
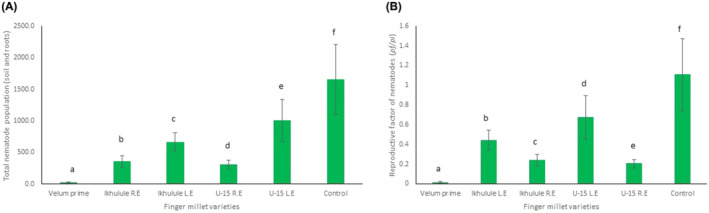
Effects of finger millet‐derived extracts on managing the root lesion nematode *Pratylenchus vandenbergae*. (A) final nematode population density in soil and roots and (B) reproductive factor of the nematodes. The values are the means of eight replicates ±SEs. Means followed by the same letter(s) are not significantly different according to the Tukey HSD test (*p* ≤ .05).

Moreover, spraying of the extracts on the leaves led to a significant decrease in the number of nematodes present in both the soil and roots of treated plants (Figure 1). When compared with the negative control, the velum prime® resulted in a 99% reduction of the nematode population. Application of Ikhulule root extract resulted in a 60% reduction in the nematode population. In contrast, Ikhulule leaf extract demonstrated a more substantial reduction, achieving a 78% decrease in nematode numbers. Similarly, for the U‐15 variety, the root extract showed a 39% decrease in the nematode population, whereas the leaf extract showed a significant 81% reduction (Figure 1A). Both leaf and root extracts of both Ikhulule and U‐15 cultivars controlled the nematode population. Additionally, all plants treated with extracts or the nematicide velum prime® had a reproductive factor (RF) of less than one. In contrast, the negative control (water‐treated plants) showed a nematode reproduction factor of greater than one (Figure 1B).

## 4 DISCUSSION

The findings of this study provide significant insights into the effectiveness of finger millet extracts in managing plant‐parasitic nematodes and promoting plant growth. The results demonstrate that extracts from the Ikhulule and U‐15 finger millet varieties possess potent nematicidal properties, achieving a mortality rate of up to 98% against *P. vandenbergae* nematodes within 72 h. This high level of efficacy highlights the potential of these finger millet extracts as a natural and sustainable alternative for nematode management in agricultural systems.

In contrast to the research by Ansari et al. (2020), which examined the effects of various plant extracts on the mortality of second‐stage juveniles (J2) of *Meloidogyne incognita*, our study demonstrated significantly higher mortality rates. Ansari et al. (2020) reported that *Achyranthes aspera* extracts at a concentration of 1000 ppm (equivalent to 1 mg/mL) led to a 16% mortality rate after 72 h. Additionally, extracts of *Alternanthera pungens* Kunth and *Amaranthus spinosus* showed mortality rates of 20% and 12%, respectively, at the same concentration. Similarly, extracts from *Dicliptera paniculata*, *Launaea procumbens*, and *Vernonia galamensis* resulted in mortality rates of 18%, 14%, and 22%, respectively, after 72 h.

Moreover, Abdullah et al. (2023) reported that leaf extracts of *Ipomoea carnea* at concentrations ranging from 250 to 1000 ppm significantly increased mortality rates of J2 of *M. incognita* over time. The study revealed a differential efficacy based on the plant organ from which the extracts were derived. Specifically, root extracts demonstrated a higher nematicidal activity compared to leaf extracts, with root extracts achieving greater mortality rates within 72 h. Conversely, our study achieved comparable results, but with the use of leaf extracts.

Botanicals can be applied through various methods, including foliar sprays, seed treatments, and soil drenching. Among these, foliar spraying is frequently preferred over soil drenching due to its significant benefits in enhancing plant growth parameters such as the number of seeds per pod and the overall yield of common bean crops. This preference highlights the potential of plant extracts in promoting plant nutrition when applied as foliar fertilizers. Such applications could prove especially beneficial in smallholder farming contexts, where issues of soil degradation are widespread (Mkindi et al., 2020).

In our study, we applied the botanicals through foliar spray and recorded a significant increase in seed weight, indicating a beneficial influence on seed development and quality. This finding is significant in the context of smallholder finger millet farmers who depend on farmer‐saved seeds for planting and household uses. High‐quality seeds are essential as they lead to more vigorous seedlings capable of withstanding biotic and abiotic stresses, ultimately resulting in higher yields (Houssard & Escarré, 1991). Afshari et al. (2011) suggested that measuring 1000‐grain weight provides a better assessment of seed quality.

Furthermore, the application of the finger millet extracts significantly decreased the number of nematodes present in both the soil and roots of treated plants, consistently maintaining a nematode reproductive factor (RF) below one. Conversely, control plants treated with water showed an RF greater than one, indicating a net increase in nematode numbers. These results indicate that finger millet extracts are effective in controlling nematode infestations. Azam et al. (2017) demonstrated the efficacy of leaf extracts of *Azadirachta indica* in reducing *M. incognita* populations in tomatoes, achieving an RF of 0.81. Similarly, Wondimeneh et al. (2013) found significant reductions in nematode densities in plants treated with extracts from *Tagetes minuta*, *Lantana camara*, and *Vernonia amygdalina*, with efficacy comparable to synthetic nematicides.

The enhanced nematicidal efficacy observed in finger millet is likely attributed to the presence of bioactive compounds that play pivotal roles in plant defense mechanisms. One such compound, phytol, a constituent of chlorophyll, serves as a significant regulator in defense responses against RKNs in *Arabidopsis thaliana*. Recent investigations have elucidated its function in inducing resistance against RKNs by modulating the ethylene signaling pathway. Upon RKN inoculation, Arabidopsis plants show an accumulation of phytol in parasitized roots, thereby inhibiting the penetration of nematodes into the roots (Fujimoto et al., 2021).

The detection of dodecanoic acid among the secondary metabolites in finger millet extracts presents an interesting discovery. Also known as lauric acid, this compound is naturally produced by crown daisy plants (*Chrysanthemum coronarium* L.) and is used in agricultural practices where these daisies are intercropped with tomatoes (*Solanum lycopersicum* L.) to manage RKNs *M. incognita*. Studies conducted by Dong et al. (2014) have demonstrated that a concentration of 4 mM dodecanoic acid effectively repels these nematodes.

Collectively, the study highlights the efficacy of finger millet‐derived extracts in managing nematodes and enhancing plant growth. These extracts offer various benefits to plants, including pathogen defense, drought tolerance, growth promotion, and physiological functions (Desmedt et al., 2020; Yates et al., 2024). For example, tannins regulate soil nitrogen dynamics (Tucuch‐Pérez et al., 2023), while saponins contribute to plant resistance against nematodes. These observations suggest that the bioactive compounds in finger millet extracts stimulate intricate physiological processes in plants, essential for defense mechanisms against pathogens. These processes likely involve hormonal pathways, such as ethylene signaling, which can be activated through foliar applications. Failure to induce these protective mechanisms renders plants vulnerable to nematode infestations, as evidenced by the susceptibility of the finger millet variety P‐224 to *P. vandenbergae* and *M. javanica* as reported by Waweru et al. (2023).

## 5 CONCLUSION

Finger millet extracts exhibit strong nematicidal properties, achieving a mortality rate of up to 98% against *P vandenbergae* nematodes. Application of these extracts leads to a significant reduction in nematode populations in soil and roots, with a reproductive factor consistently below one, indicating effective nematode control. The study attributes the enhanced nematicidal effect to high concentration of bioactive compounds, notably phytol, associated with resistance against RKNs in other plants. Additionally, finger millet‐derived extracts promote the growth of finger millet plants, enhancing seed weight and potentially improving seed quality. These findings suggest that finger millet extracts offer a natural solution for nematode management and broader agronomic benefits, enhancing overall plant health and productivity.

## CONFLICT OF INTEREST STATEMENT

The authors declare that they have no conflict of interest.

## Supporting information


Data S1.

## Data Availability

The data that support the findings of this study are available in the supplementary material of this article.
